# Gene Expression Network Reconstruction by LEP Method Using Microarray Data

**DOI:** 10.1100/2012/753430

**Published:** 2012-12-23

**Authors:** Na You, Peng Mou, Ting Qiu, Qiang Kou, Huaijin Zhu, Yuexi Chen, Xueqin Wang

**Affiliations:** School of Mathematics & Computational Science, Sun Yat-Sen University, Guangzhou, Guangdong 510275, China

## Abstract

Gene expression network reconstruction using microarray data is widely studied aiming to investigate the behavior of a gene cluster simultaneously. Under the Gaussian assumption, the conditional dependence between genes in the network is fully described by the partial correlation coefficient matrix. Due to the high dimensionality and sparsity, we utilize the LEP method to estimate it in this paper. Compared to the existing methods, the LEP reaches the highest PPV with the sensitivity controlled at the satisfactory level. A set of gene expression data from the HapMap project is analyzed for illustration.

## 1. Introduction

Genes on the chromosomes behave interactively controlling the gene expression profiles of a cluster of genes, and their own expressions are in turn regulated by a bundle of genes. Exploring the gene expression regulatory network is essentially important to understand the progress of complex diseases, find the causal genes, and develop new drugs. In the past decades, the development of microarray technology allows us to measure the expression levels of tens of thousands of genes simultaneously, providing an opportunity to study the complex relationships among genes. In order to reconstruct the gene expression network, for any two particular genes, the conditional independence given all other genes needs to be investigated.

Because of the convenience of describing the interactions among variables, the graphical models become a common choice to study the relationships between variables, including but not limited to Boolean network [1], Bayesian network [2–4], autoregression model [5], and graphical Gaussian model [6]. However, the statistical inference on the independence is not easy. Under the Gaussian assumption, the independence is identical to being uncorrelated, and the conditional dependence between variables is able to be represented by the partial correlation coefficient matrix. When the number of observations *n* is equal or greater than the number of variables *p*, [7] mentioned two ways to estimate the partial correlation coefficient matrix in the graphical Gaussian model. If *n* < *p*, neither of these two ways is applicable due to the singular matrix.

As a typical high-dimensional data, there are usually not many available chips, while a great number of genes are included in the microarray data analysis. Fortunately, more and more studies [8–10] showed that the gene expression network is sparse, which means, for a particular gene, it only interacts with a few other genes. This fact implies that the majority entries of the partial correlation coefficient matrix are zero. To efficiently explore the sparsity and identify non-zero entries, the penalized linear regression is established where the sum of squared residuals (SSR) plus a penalty term is minimized, and has been widely used to estimate the sparse partial correlation coefficient matrix to reconstruct the gene expression network using microarray data [7, 11].

The most pioneering penalized linear regression method, the least absolute shrinkage and selection operator (LASSO) proposed by [12], utilizes the *L*_1_ penalty to shrink the estimate which is close to zero from non-zero to zero, but it shrinks the estimates for parameters farther away from zero more severely, leading to a substantial bias. The authors in [13] indicated that LASSO may cause a bias even in a simple regression and suggested the smoothly clipped absolute deviation (SCAD) method, where a nonconcave penalty term with desirable statistical properties, such as unbiasedness, sparsity, and continuity, was introduced. However, the SCAD penalty is not smooth, resulting in the optimization problem being complicated. Upon this, [14] proposed the Laplace error penalty (LEP) method with a penalty which is unbiased, sparse, continuous, and almost smooth.

In this paper, we will apply the LEP method to reconstruct the gene expression network, and compare it to LASSO and SCAD in the performance of estimating the partial correlation coefficient matrix. The paper is structured as follows. In Section 2, the LASSO, SCAD, and LEP methods will be briefly described. In Section 3, we will report the results of simulations and a real data analysis. A short discussion is given in Section 4.

## 2. Methods

The graphical Gaussian model, or GGM for abbreviation, is an undirected graphical model. Let **X** = (*X*_1_,…, *X*_*p*_)′ indicate a *p*-dimensional random variable, subject to the multivariate normal distribution *N*(***μ***, Σ), where ***μ*** is the mean vector and Σ is the variance-covariance matrix. Given *n* samples from *N*(***μ***, Σ), (*x*_*ij*_)_*p*×*n*_, the partial correlation coefficient matrix (*ρ*_*ij*_)_*p*×*p*_, which reflects the conditional dependence between different components of **X**, could be estimated by ${\hat{\rho }}_{ij}=\mathrm{sign}\left({\hat{\beta }}_{ij}\right)\sqrt{{\hat{\beta }}_{ij}{\hat{\beta }}_{ji}}$, where ${\hat{\beta }}_{ij}$ is the estimator for *β*_*ij*_ in the linear regression model

(1) $$\begin{array}{c} X_{ij}=\sum_{1\leq k\neq i\leq p}\beta_{kj}X_{kj}+\epsilon_{ij},\;i=1,2,\ldots ,p;\;\;j=1,2,\ldots ,n, \end{array}$$

*ϵ*_*ij*_, *i* = 1,2,…, *p* and *j* = 1,2,…, *n*, are independent and identically distributed, and independent of **X**, and sign(*x*) is an indicator function, being −1, 0, or 1 when *x* is smaller, equal, or greater than 0, respectively. For the “small N large P” problem, instead of the classical least square optimization, the objective function

(2) $$\begin{array}{c} \sum_{i=1}^{p}\sum_{j=1}^{n}{\left(X_{ij}-\sum_{k\neq i}\beta_{kj}X_{kj}\right)}^{2}+\sum_{i=1\;}^{p}\sum_{1\leq j\neq i\leq N}p_{\lambda }\left(\beta_{ij}\right) \end{array}$$

is minimized to get the estimator for *β*_*ij*_, ${\hat{\beta }}_{ij}$, where *p*_*λ*_(·) indicates a penalty function on the parameters. The formula *p*_*λ*_(·) is essentially important. It not only determines the way to shrink the estimators, but also directly affects the complexity of the optimization algorithm. A good penalty function should have several desirable statistical properties, unbiasedness, sparsity, continuity [13], and smoothness [14].

The LASSO, proposed by [12], has the penalty *p*_*λ*_(*β*) = *λ*|*β*|. Although it succeeded in many applications of variable selection, it shrinks the estimates of larger parameters more significantly than that of the smaller parameters, causing a substantial bias. The SCAD penalty function, suggested by [13], has the derivative *p*_*λ*_′(*β*) = *λ*{*I*(|*β*| ≤ *λ*) + (*λα*−|*β*|)_+_ *I*(|*β*| > *λ*)/(*λ*(*α* − 1))}. Beside the sparsity and continuity, it gains the unbiasedness but loses the smoothness. The SCAD penalty is made of piecewise quadratic splines, making the optimization of (2) complicated. To overcome this problem, Wen et al. [14] proposed the LEP with penalty term

(3) $$\begin{array}{c} p_{\lambda }\left(\beta \right)=\lambda \left(1-\exp\left(-\frac{\left|\beta \right|}{\kappa }\right)\right), \end{array}$$

where *λ* and *κ* > 0 are two tuning parameters.

The LEP penalty not only satisfies the unbiasedness, sparsity, and continuity, but also is an almost smooth function. It emphasizes the smoothness and complexity, since the smooth function is more stable, and the complexity of optimization algorithm highly depends on the complexity of *p*_*λ*_(·), which determines whether the proposed method could be widely applied, especially in the high-dimensional data situations. In order to solve the optimization problem, [14] extended the block coordinate gradient descent (BCGD) algorithm [15] and provided a faster computing algorithm, as will be shown in the simulation studies. For the details of the LEP method and the optimization algorithm, please refer to [14].

## 3. Results

### 3.1. Simulations

Suppose there are *n* microarray chips and *p* genes, then *n* × *p* equations with *p* × (*p* − 1) parameters are involved in (1). When *p* is fixed, increasing/decreasing *n* would increase/decrease the number of equations but the number of parameters would remain the same. In this case, the penalized linear regression, including LEP, LASSO and SCAD, performed as expected that is, their estimates became more or less accurate as *n* became larger or smaller (results not shown here). Therefore, in the following simulations, we fixed *n* = 120 and only varied *p* = 10 or 20.

In order to fully evaluate the performances of LEP, LASSO, and SCAD in different situations, four scenarios were set up. In each scenario, a covariance matrix Σ of size *p* × *p* was generated, and *n* random vectors of dimension *p* were sampled from the multivariate normal distribution *N*(0, Σ) independently. The partial correlation coefficient matrix was then estimated from the sampled data. We fixed Σ and made 100 repetitions in each scenario to get the average of the estimates for fair comparison. In the first two scenarios *p* = 10, and *p* = 20 in scenario 3 and 4. Two data generating procedures used in [11] were employed to generate the covariance matrix Σ. In scenario 1 and 3, the (*i*, *j*)-element of Σ, *σ*_*ij*_ = exp(−*a*|*s*_*i*_ − *s*_*j*_|), where *a* = 2 and *s*_1_ < *s*_2_ < ⋯<*s*_*p*_ were generated by setting *s*_*i*_ − *s*_*i*−1_, following a uniform distribution *U*(0.5,1). In scenario 2 and 4, a sparse precision matrix *Ω* was generated as proposed in [16], and Σ = *Ω* ^−1^.

The partial correlation coefficient matrix was estimated by LEP, LASSO or SCAD, respectively, in each scenario. To evaluate the performances of different methods, the sensitivity which is the fraction of “true non-zero and also estimated non-zero parameters” to “true non-zero parameters” and PPV which is the fraction of “true non-zero and also estimated non-zero parameters” to “estimated non-zero parameters” were calculated. Furthermore, the *F*_1_  score = 2 · sensitivity · PPV/(sensitivity + PPV) was also presented. The results in four scenarios were listed in Table 1.

As the number of genes *p* increases, the number of parameters to be estimated increases rapidly. Due to the sparsity of partial correlation coefficient matrix, the number of true zero parameters increases much more than that of true non-zero parameters, causing the chance of estimating a zero parameter to be non-zero increases more than that of estimating a non-zero parameter to be zero. As presented in Table 1, although the sensitivity of LEP did not change significantly as *p* increasing from 10 to 20, its PPV reduced obviously from ~90% in scenario 1 and 2 to ~80% in scenario 3 and 4. The LASSO and SCAD showed similarly. Note that beside the penalty term, the performances of different methods also depend on the true value of covariance matrix Σ, which was generated at the beginning of each scenario.

Across all the scenarios, although LASSO reached the highest sensitivity, its PPV was far lower than that of SCAD and LEP, which means that LASSO could identify more gene regulatory relationships, but there might be many false positives. Among these three methods, LEP achieved the highest PPV with its sensitivity controlled at similar level to that of SCAD. Its *F*_1_ score also reached the highest value in scenario 1, 3, and 4. More importantly, using the algorithm proposed by [14], LEP was the fastest, whose computation time was almost 1/18, 1/10, 1/7 and 1/9 of LASSO and 1/5, 1/4, 1/3, and 1/3 of SCAD in four scenarios, respectively.

**Figure 1:**
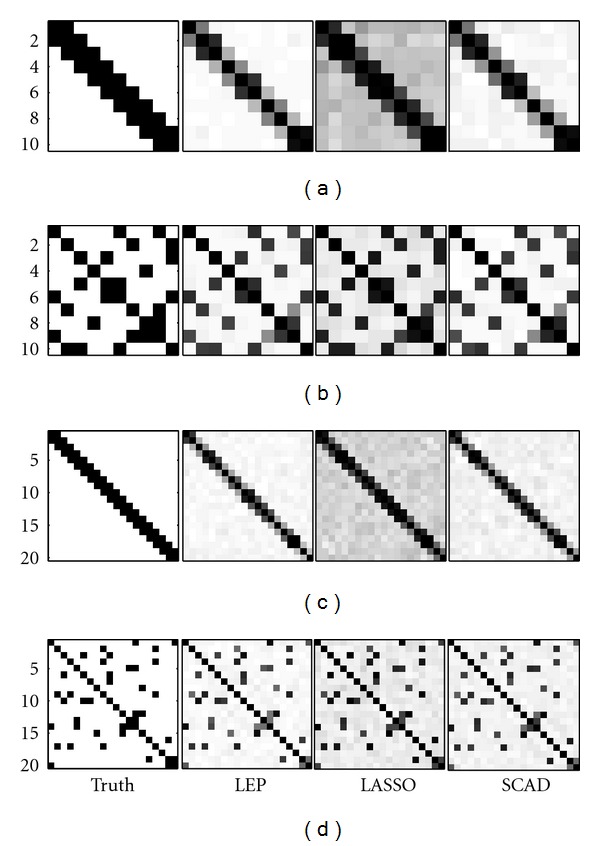
The relative frequency matrices in four scenarios of the simulation studies. The first, second, third and forth rows correspond to scenario 1, 2, 3 and 4, respectively.

For intuitive illustration, we also plotted the relative frequency matrix for each method in each scenario, where the (*i*, *j*)-element indicates the relative frequency of non-zero estimates among 100 repetitions. The darker the color is, the higher the frequency of non-zero estimates is. The true partial correlation coefficient matrix was shown in the first panel of each row in Figure 1. From Figure 1, we can see that the color of LASSO is significantly darker than others, especially the truth, which means that LASSO estimated many true zero parameters to be non-zero, resulting in many false positives. Comparing to LASSO, the SCAD plot became much closer to the truth and LEP made a further improvement upon the SCAD plot.

### 3.2. A Real Data Example

In this section, the publicly available gene expression dataset GSE6536 (http://www.ncbi.nlm.nih.gov/geo/query/acc.cgi?acc=GSE6536) was investigated. There are gene expression values of 47,294 human transcripts from 270 HapMap individual samples [17], including 30 Caucasian trios of northern and western European background (CEU), 30 Yoruba trios from Ibadan, Nigeria (YRI), 45 unrelated individuals from Beijing, China (CHB), and 45 unrelated individuals from Tokyo, Japan (JPT). After the microarray data were log2-transformed and background corrected, within and across the population normalized, the gene expression values were saved in a matrix for further analysis, which also could be downloaded from the website mentioned above.

Frommlet et al. [18] listed 44 genes which are significantly differentially expressed across individual samples. Since the platform Sentrix Human-6 Expression BeadChip used in this experiment was publicly available in 2005, so far the gene annotation database has been updated a lot. Of those 44 genes, 14 either were found to be pseudogenes or have been removed as a result of standard genome annotation processing and therefore were excluded from our following analysis. The partial correlation coefficient matrix of the rest 30 genes were estimated using 270 samples data. In the graphical model, one non-zero estimate off diagonal in the partial correlation coefficient matrix corresponds to one edge connecting different genes on the graph. The number of edges connected with each gene was listed in Table 2.

As shown in Table 2, LASSO identified 76 edges between 30 genes, SCAD found 21 and LEP reported 13. The LASSO recognized that almost all of the genes interacted with others and identified much more edges than SCAD or LEP. To compare the performances of different methods, we only focus on the important genes which carry the most or secondly most number of edges. For LASSO, there is 1 such important gene with 9 edges and 4 with 8 edges each. The SCAD found 2 important genes with 4 edges each and 4 with 3 edges each. The LEP identified 4 with 3 edges each and 4 with 2 edges each.

For the important genes recognized by LASSO, none of them were taken to be important by SCAD or LEP. According to the gene functions described in Table 2, although these genes have very important functions, they usually accomplish these functions together with many others genes, and once they could not be normally expressed, these functions could be completed by other genes, then this would not significantly affect the gene expression in the network. On the contrary, the genes identified by SCAD or LEP usually have unique gene function, which could not be recovered by other genes once they are expressed abnormally, resulting in the irregular expression in the network.

Beside those 5 common genes identified by both of LEP and SCAD, LEP found 3 more exclusive genes and SCAD found 1 more exclusive gene. Those 3 LEP exclusive genes not only play a key role in the cellular mechanism, but also have very close relationships with other genes. Among them, PRPF8 (gene 16) is a component of both U2- and U12-dependent spliceosomes, which removes the vast majority of introns (more than 99%) in mammals [19, 20]. DDR1 (gene 21), one of the receptor tyrosine kinases, is important in the communication between the cells and their microenvironments and gets involved in many cellular activities, like growth, differentiation, and metabolism [21]. UBA7 (gene 26), widely expressed in a variety of cell types, belongs to the ubiquitin conjugation pathway, which is of fundamental and central importance [22]. However, the SCAD exclusive gene, TARDBP (gene 19), although plays an important role in modulating HIV-1 gene expression; it only represses transcription from the HIV-1 long terminal repeat, no other transcription from other promoters [23]. Due to this fact, it should not interact heavily with other genes, as the LEP concluded.

## 4. Discussion

In this paper, we applied the LEP method to estimate the partial correlation coefficient matrix to reconstruct the gene expression network. Comparing to the existing methods, for example, LASSO and SCAD, LEP reached the highest PPV, and its sensitivity was controlled at the similar level as SCAD. As seen from the relative frequency matrix plot in the simulation studies, LEP showed the superiority in exploring the sparsity of the partial correlation coefficient matrix.

There are two tuning parameters in the LEP penalty function. We used the EBIC criteria [24] to select the approximate values for parameters. But as seen from the simulation results (not shown here), any combination of *κ* and *λ* which satisfy some certain function relation would return very close estimation results. Therefore, we only need to vary one of *κ* and *λ* and keep the other a constant for parameter choosing. As in the real data analysis, we set *κ* = 0.01 and vary *λ*.

## Figures and Tables

**Table 1:** The sensitivity, PPV, and *F*_1_ values in four scenarios of the simulation studies.

	PPV	Sensitivity	*F*_1_	SSE	Time (s)
		Scenario 1: *P* = 10			

LEP	0.934	0.708	0.805	1075.313	0.121
LASSO	0.602	0.908	0.724	1053.937	2.184
SCAD	0.891	0.727	0.801	1070.202	0.594

		Scenario 2: *P* = 10			

LEP	0.926	0.826	0.873	1185.403	0.259
LASSO	0.833	0.916	0.873	1215.098	2.507
SCAD	0.932	0.828	0.877	1191.352	0.933

		Scenario 3: *P* = 20			

LEP	0.778	0.707	0.741	2112.376	2.431
LASSO	0.467	0.868	0.607	2100.014	16.976
SCAD	0.693	0.741	0.716	2089.248	6.247

		Scenario 4: *P* = 20			

LEP	0.831	0.834	0.832	2351.997	2.763
LASSO	0.667	0.910	0.770	2380.255	23.792
SCAD	0.735	0.852	0.789	2298.897	7.193

**Table 2:** The number of edges connected with each gene in GSE6536 data example.

No.	Gene	LEP	LASSO	SCAD	Gene functions
1	ABCF1	0	9	1	ATP-binding cassette
2	EIF3D	0	8	1	Eukaryotic translation initiation factor 3
3	SRP14	0	8	1	Signal recognition particle 14 kDa (homologous Alu RNA-binding protein)
4	RPL28	0	8	0	Ribosomal protein L28
5	EIF3F	0	8	0	Eukaryotic translation initiation factor 3
6	CYP2A6	3	7	3	Cytochrome P450
7	RPL35	0	7	2	Ribosomal protein L35
8	GDI2	0	7	1	GDP dissociation inhibitor 2
9	RPL11	0	7	1	Ribosomal protein L11
10	GAS6	3	6	3	Growth arrest-specific 6
11	DAD1	1	6	2	Defender against cell death 1
12	RPL21	0	6	1	Ribosomal protein L21
13	EPHB3	3	5	3	EPH receptor B3
14	MMP14	2	5	4	Matrix metallopeptidase 14 (membrane inserted)
15	ESRRA	2	5	3	Estrogen-related receptor alpha
16	PRPF8*	2	5	0	PRP8 pre-mRNA processing factor 8 homolog (*S. cerevisiae*)
17	HSPA6	1	5	2	Heat shock 70 kDa protein 6 (HSP70B′)
18	PARK7	1	5	2	Parkinson protein 7
19	TARDBP	0	5	4	TAR DNA-binding protein
20	SEPT2	0	5	0	Septin 2
21	DDR1*	3	4	0	Discoidin domain receptor tyrosine kinase 1
22	TRADD	1	4	2	TNFRSF1A-associated via death domain
23	EIF4G2	0	4	1	Eukaryotic translation initiation factor 4 gamma
24	CAPNS1	0	4	0	Calpain, small subunit
25	PLD1	1	3	2	Phospholipase D1
26	UBA7*	2	2	0	Ubiquitin-like modifier activating enzyme 7
27	CYP2E1	0	2	1	Cytochrome P450
28	FNTB	1	1	1	Farnesyltransferase
29	GUCA1A	0	1	1	Guanylate cyclase activator 1A (retina)
30	CCL5	0	0	0	Chemokine (C-C motif) ligand 5

*Indicates LEP exclusive genes.
